# Supplementary material for: Whole-genome sequence analysis of high-level penicillin-resistant strains and antimicrobial susceptibility of Neisseria gonorrhoeae clinical isolates from Thailand
Source: PLoS One. 2022 Jul 29;17(7):e0271657. doi: 10.1371/journal.pone.0271657 (PMC9337635; doi:10.1371/journal.pone.0271657)
Supplement: S1 Table — (DOCX) [file pone.0271657.s001.docx]

**S1 Table. Genome sequencing and assembly statistics for 3 *Neisseria gonorrhoeae* strains with high-level resistance to penicillin.**

| **Parameter** | **CT530** | **CT532** | **CT602** |
| --- | --- | --- | --- |
| **SRA accession no.** |  |  |  |
| Illumina | [SRR10861752](https://trace.ncbi.nlm.nih.gov/Traces/sra/?run=SRR10861752) | [SRR10861750](https://trace.ncbi.nlm.nih.gov/Traces/sra/?run=SRR10861750) | [SRR10861748](https://trace.ncbi.nlm.nih.gov/Traces/sra/?run=SRR10861748) |
| ONT | [SRR10861751](https://trace.ncbi.nlm.nih.gov/Traces/sra/?run=SRR10861751) | [SRR10861749](https://trace.ncbi.nlm.nih.gov/Traces/sra/?run=SRR10861749) | [SRR10861747](https://trace.ncbi.nlm.nih.gov/Traces/sra/?run=SRR10861747) |
| **Total no. of reads** |  |  |  |
| Illumina | 10316946 | 9796802 | 11656528 |
| ONT | 98030 | 102879 | 229766 |
| **Average read length** |  |  |  |
| Illumina | 149.94 | 149.96 | 149.95 |
| ONT | 4985.70 | 4594.49 | 4866.11 |
| **Longest read length** |  |  |  |
| Illumina | 150 | 150 | 150 |
| ONT | 50027 | 47862 | 50804 |
| **N50 (bp)** |  |  |  |
| Illumina | 150 | 150 | 150 |
| ONT | 5046 | 4722 | 5731 |
| **GenBank accession no.** |  |  |  |
| Chromosome | [CP048254](https://identifiers.org/resolve?query=insdc:CP048254) | [CP048250](https://identifiers.org/resolve?query=insdc:CP048250) | [CP048246](https://identifiers.org/resolve?query=insdc:CP048246) |
| Plasmids | [CP048255](https://identifiers.org/resolve?query=insdc:CP048255), [CP048256](https://identifiers.org/resolve?query=insdc:CP048256), [CP048257](https://identifiers.org/resolve?query=insdc:CP048257) | [CP048251](https://identifiers.org/resolve?query=insdc:CP048251), [CP048252](https://identifiers.org/resolve?query=insdc:CP048252), [CP048253](https://identifiers.org/resolve?query=insdc:CP048253) | [CP048247](https://identifiers.org/resolve?query=insdc:CP048247), [CP048248](https://identifiers.org/resolve?query=insdc:CP048248), [CP048249](https://identifiers.org/resolve?query=insdc:CP048249) |
| Genome size (bp) | 2169323 | 2165075 | 2162702 |
| Plasmid size (bp) | 42003, 5111, 4153 | 42001, 5155, 4207 | 39072, 5111, 4207 |
| Genome coverage (×) | 938.4 | 896.9 | 1325.2 |
| GC content (%) | 52.6 | 52.7 | 52.7 |
