# Supplementary material for: Whole-genome sequence analysis of high-level penicillin-resistant strains and antimicrobial susceptibility of Neisseria gonorrhoeae clinical isolates from Thailand
Source: PLoS One. 2022 Jul 29;17(7):e0271657. doi: 10.1371/journal.pone.0271657 (PMC9337635; doi:10.1371/journal.pone.0271657)
Supplement: Attachment — Submitted filename: Reviewer comments PLOS 1.docx [file pone.0271657.s003.docx]

Reviewer comments:

Whole-genome sequence analysis of high-level penicillin-resistant strains and 2 antimicrobial susceptibilities of Neisseria gonorrhoeae clinical isolates from Thailand

Abstract/ Introduction

Adding statement to justify the importance of study regionally and globally;

As this study is from Thailand (the study region), giving a general impact of gonococcal infection and the effect of AMR in the study region would elaborate on the relevance and importance of the study locally and globally.

Justification of sample size/patient recruitment

Justification of only 03 strains analysis

Abstract line 32; mtrR were performed. Need to define mtrR

Line 38: notifying the significance of the genes/ genetic characteristics will highlight the importance of the study further

46, 47 The gonococcal population in Thailand showed high susceptibility to all antimicrobials 47 recommended for gonorrhoea treatment.

But the line 37: All isolates were susceptible to spectinomycin, can you clarify the comment in lines 46,47

48: expanded and enhanced surveillance of antimicrobial susceptibility 49 and study of genetic resistance determinants are essential to improve treatment guidelines.

Can you clarify why this is needed by considering the actual prevalence of gonococcal infection prevalence in Thailand?

Introduction

Line 74: What is the significance of gonococcal infection prevalence in Thailand?

Line 90; (M182T) should be referred to as strain?

Line 122: should bring to earlier and also compare with global statistics to show why this study is important to Thailand

Line 155: do the participants considered have received the antibiotic treatment before the study?

What is the statistical method used for Patient data and Prevalence and penicillin susceptibility of PPNG isolate

Line 255: Do the study participants give their consent if so mention this in this section.

Line 181: interpretative criteria of the guidelines; can you clarify further with regards to MIC

Line 183; What is the coverage of the N gonorrhoea genome? Are the AMR determinants considered located in short or long read sequences?. Justify the usage of both sequencing methodologies.

Line 465 what is the significance of the MLST ST8143., clarify

Line 528: is there a positive correlation between the MIC and the corresponding genetic determinants/ it is not very clear

Line 535: how to justify the statement; We provide the first genomic analysis of all plasmids in N. gonorrhoeae strains from Thailand. Clarify it further

Line 555: it is worth mention the previous antibiotic treatment data is also not available

Is the risk factors referring to the patients?
